# Potential of Alkaloids from *Zanthoxylum nitidum* var. *tomentosum* in Treating Rat Rheumatoid Arthritis Model and Validation of Molecular Mechanisms

**DOI:** 10.3390/cimb47080661

**Published:** 2025-08-15

**Authors:** Yuanle Shen, Linghui Zou, Yinggang Zeng, Ting Xia, Zhenjie Liu, Kaili Hu, Liuping Wang, Jianfang Feng

**Affiliations:** 1 School of Pharmacy, Guangxi University of Chinese Medicine, Nanning 530200, China; shenyuanle2022@163.com (Y.S.); zengyinggang2016@163.com (Y.Z.); xiating0226@163.com (T.X.); rainman982@126.com (Z.L.); rousel@126.com (L.W.); 2 School of Pharmacy, Shanghai University of Traditional Chinese Medicine, Shanghai 201203, China; linghuibox@163.com; 3 Guangxi Engineering Technology Research Center of Advantage Chinese Patent Drug and Ethnic Drug Development, Guangxi University of Chinese Medicine, Nanning 530200, China

**Keywords:** *Zanthoxylum nitidum* var. *tomentosum*, alkaloids, rheumatoid arthritis, network pharmacology, mechanism validation

## Abstract

Background: Rheumatoid arthritis (RA) is a chronic inflammatory disorder characterized by synovial hyperplasia and joint destruction. Previous studies have demonstrated that the alkaloids of Rushanhu (ARSHs), the dried root and stem of Zanthoxylum nitidum var. tomentosum, exhibit favorable therapeutic effects on RA, and this study aims to investigate the underlying molecular mechanisms involved. Methods: A complete Freund’s adjuvant (CFA)-induced arthritis model in male SD rats (*n* = 64) was used to evaluate ARSHs. Groups included control, model, methotrexate (MTX), and ARSH-treated. Therapeutic effects were assessed via arthritis index, paw swelling, and serum cytokines (IL-1β, IL-6, IL-17A). Network pharmacology identified bioactive alkaloids and core targets, validated by molecular docking. In vitro mechanisms (proliferation, apoptosis, signaling pathways) were examined in MH7A synovial cells. Results: ARSHs significantly attenuated joint inflammation and damage in CFA rats (* *p* < 0.01 vs. model), reducing pro-inflammatory cytokines. Fifteen alkaloids (e.g., dihydrochelerythrine, magnoflorine) and 24 targets (e.g., SRC, STAT3, MAPK3) were prioritized. Molecular docking confirmed strong binding (binding energy < −7.0 kcal/mol). In vitro, ARSHs suppressed MH7A proliferation and induced apoptosis via Bcl-2/Bax dysregulation and the inhibition of SRC/STAT3/MAPK3 phosphorylation. Conclusions: ARSHs mitigate RA pathogenesis by targeting the SRC/STAT3/MAPK3 signaling axis in synovial cells. This study provides mechanistic validation of ARSHs as multi-target phytotherapeutic agents against inflammatory arthritis.

## 1. Introduction

Rheumatoid arthritis (RA) is a common, chronic autoimmune disease of undetermined etiology, with its main pathologic manifestations being synovial hyperplasia, inflammatory cell infiltration, and cartilage erosion. The clinical manifestations are pain and swelling at the joints, or worse, joint deformity or severe disability [1]. Currently, RA therapeutic drugs mainly consist of nonsteroidal anti-inflammatory drugs (NSAIDs), drugs to improve rheumatic conditions (DMARDs), glucocorticoids (GCs), and biologics [2]. However, these drugs are expensive and associated with adverse effects such as cardiovascular and gastrointestinal bleeding, liver and kidney toxicity, and growth inhibition [3,4,5,6,7]. Chinese ethnomedicine and Chinese herbal medicine have the special advantages of multi-components, multi-targets, and low adverse effects. In addition, many active ingredients and their action mechanisms have been confirmed in clinical and experimental studies, with promising potential for application [8,9].

The Yao ethnomedicine Rushanhu (RSH) is made from the dried roots and stems of *Zanthoxylum nitidum* (*Roxb.*) *DC.* var. *tomentosum* Huang of the family Rutaceae [10], which is a highly essential component in many traditional Yao ethnomedicine prescriptions. Yao ethnomedicine considers that drugs like RSH can be used to try to treat serious clinical diseases. According to the theory of traditional Chinese medicine, RSH has the efficacy of clearing heat and detoxifying [11] and subduing swelling and relieving pain; RSH can be used in the treatment of rheumatism [12] and rheumatoid arthritis [13]. Modern pharmacological studies have indicated that the root of RSH is used as medicine and has pharmacological efficacy, including anti-inflammatory [14], antifungal [15], antibacterial [16], antitumor, and anticancer effects [17]. However, as a commonly used anti-RA Yao ethnomedicine, research on the material basis of the anti-RA pharmacological effect of RSH is still weak. Therefore, it is necessary to conduct more in-depth studies on the potential mechanisms of RSH against RA.

Herein, our team screened the therapeutic effects of different extracts from RSH on RA through an RA rat model and found that the alkaloids of RAH (ARSHs) exhibited the best anti-RA activity. Further, we predicted the active components and targets of anti-RA ARSHs by network pharmacology and molecular docking techniques. Finally, we experimentally validated the predicted targets using the MH7A cell model and found that the anti-RA mechanisms of ARSHs were closely related to the down-regulation of Bcl-2, up-regulation of Bax, and inhibition of SRC/STAT3/MAPK3 expression. These findings may provide a preliminary scientific basis for the study of the anti-RA effects of RSH and provide a reference for the development of anti-RA drugs.

## 2. Materials and Methods

### 2.1. Experimental Materials

#### 2.1.1. Drugs and Reagents

The RSH samples were identified as the dried roots and stems of *Zanthoxylum nitidum* (*Roxb.*) *DC.* var. *tomentosum* Huang by Prof. Wei Songji and Mr. Zhu Yilin. Enzyme-linked immunosorbent assay (ELISA) kits for IL-6, IL-10, and TNF-α were purchased from Thermo Fisher Scientific (China) Co., Ltd. (Wuhan, China) and Wuhan Eliot Biotechnology Ltd. Magnoflorine (purity: 99.02%) (Wuhan, China) and dihydrochelerythrine (purity: 99.21%) was purchased from Chengdu Manstar Biotechnology Ltd. (Chengdu, China). Nitidine chloride (purity: 91.00%) was purchased from the China Academy of Food and Drug Administration (Beijing, China). Rabbit anti-SRC, rabbit anti-STAT3, rabbit anti-MAPK3, rabbit anti-BAX, rabbit anti-BCL-2, horseradish peroxidase (HRP)-conjugated goat anti-rabbit secondary antibody, and HRP-conjugated goat anti-mouse secondary antibody were purchased from Wuhan Xavier Biotechnology Ltd. (Wuhan, China). Experimental reagents and instruments used in this study are listed in Supplementary Tables S1–S7.

#### 2.1.2. Animals

Six-week-old SPF-grade SD male rats (180–220 g) were purchased from Hunan Slake Kingda Laboratory Animal Co., Ltd. (Changsha, China) with license number SCXK (Xiang) 2019-0004. The rats were kept in an environment with 50% relative humidity, a temperature of (22 ± 1) °C, and light control for 12 h of alternating light and darkness, and were fed with standard feed and given free access to sterilized water. The animal experiments were approved by the Ethics Committee of Guangxi University of Traditional Chinese Medicine, in accordance with the principles of animal protection, animal welfare, and ethics, with approval number DW20220526-114 (26 May 2022).

#### 2.1.3. Cells

MH7A human rheumatoid arthritis fibroblasts were purchased from Bei Na Biotechnology Co., Ltd. (Xinyang, China) The cells were routinely cultured in DMEM high-glucose medium containing 1% penicillin and streptomycin and 10% FBS in an incubator at 37 °C with 5% CO_2_.

### 2.2. In Vivo Screening Study of Anti-RA Active Extracts of RSH

#### 2.2.1. Preparation of Different Extracts from RSH

Preparation of total alkaloid extract from RSH. The total alkaloid extract was prepared using the acid extraction and alkaline precipitation method. A total of 8.5 kg of coarse powder from the roots and stems of RSH was poured into the percolation tank in small batches and spread evenly. A total of 10 volumes of 0.8% dilute hydrochloric acid were added for maceration for 3 days, with stirring conducted twice a day. Percolation was carried out at an appropriate flow rate, and the extracts were collected and combined. Ammonia water was added to adjust the pH to 10, and the alkaloid precipitate was obtained by suction filtration. Meanwhile, the filtrate was concentrated under reduced pressure, and then chloroform was used to extract the alkaloids into the chloroform layer based on their liposolubility, resulting in the total alkaloid extract. The solvent was recovered by concentration under reduced pressure, and the concentrated total alkaloid extract ointment was 1286.52 g, with an extraction yield of 15.14%. The alkaloid group (alkaloid-enriched fraction) specifically refers to the total alkaloid fraction extracted via acid–base precipitation and chloroform partitioning (enriched in alkaloids, as confirmed by UPLC-ESI/Q-TOF-MS/MS).

Preparation of petroleum ether, ethyl acetate, n-butanol, and water extract from RSH. The 8.5 kg of coarse powder from the roots and stems of RSH was poured into the percolation tank in small batches and spread evenly. A total of 10 volumes of 70% ethanol were added for maceration for 3 days, with stirring conducted twice a day. Percolation was performed at an appropriate flow rate, and the extracts were collected, deslagged, and combined. The solvent was recovered by concentration under reduced pressure until there was no alcohol odor, obtaining the total ethanol extract ointment. The systematic solvent method was used to separate the total ethanol extract of RSH into fractions of different polarities: distilled water was added to the total ethanol extract ointment at a ratio of 1:2 for suspension, and the above suspension was successively extracted multiple times with petroleum ether, ethyl acetate, and n-butanol. The solutions obtained after extraction were combined and concentrated, respectively, to obtain the ointments of the petroleum ether fraction, ethyl acetate fraction, n-butanol fraction, and water extract of RSH, with extraction yields of 0.46%, 2.08%, 4.43%, and 5.82%, respectively. The obtained ointments were stored in a refrigerator at 4 °C for later use. Other groups (petroleum ether, ethyl acetate, n-butanol, and water extract) were non-alkaloid solvent fractions.

Based on the clinical dose of RSH (15 g/60 kg for humans), the equivalent dose for rats was converted to 1.5 g/kg by the body surface area conversion method (coefficient of 6.17). After the pre-experimental dose exploration, 6 g/kg (4 times the equivalent dose) was finally selected as the administered dose. A test solution of 0.6 g/mL (containing 0.6 g/mL of raw drug) was prepared at 0.5% CMC-Na.

#### 2.2.2. Construction of RA Rat Model and Drug Administration

Sixty-four SD male rats (the sample size (*n* = 8 per group) was chosen based on feasibility within the study’s resource constraints and alignment with similar pilot studies in the field) were randomly divided into a blank group; a model group; a methotrexate (MTX) as a positive drug control group; and a group of different extracts, such as alkaloids (petroleum ether, ethyl acetate, n-butanol, and water extract). The RA model was induced by the intradermal injection of 0.2 mL of complete Freund’s adjuvant (CFA) into the right hind toe of each group. After successful modeling, the blank group and the model group were gavage-administered with 0.5% CMC-Na solution. The MTX group was dosed with 0.5 mg/kg (twice/week), and the groups with different extracts were gavage-administered for 22 days at 6 g/kg/day as designed. The arthritis index (unilateral hindfoot score × 4, Table 1) was recorded every 4 days starting from the 2nd day after modeling, the thickness of the right hindfoot metatarsal was measured at the same time, and the degree of swelling was calculated (thickness after modeling–thickness before modeling). Protocol flow chart for drug administration in the RA rat model (Figure S1).

#### 2.2.3. Serum Collection and Measurement of Inflammatory Factors

The rats were anesthetized by the intraperitoneal injection of 20% urethane at 1 g/kg. After successful anesthesia, blood was collected from the abdominal aorta, and the serum was separated by centrifugation at 3000 rpm for 10 min at 4 °C. The levels of IL-1β, IL-4, IL-6, IL-10, and IL-17A in serum were determined using the corresponding ELISA kits.

### 2.3. Pharmacology of Anti-RA Network and Molecular Docking Prediction of Alkaloids from RSH (ARSHs)

#### 2.3.1. Determination of Active Components in ARSHs

The chemical compositions of ARSHs were clarified using ultra-performance liquid quadrupole time-of-flight mass spectrometry (UPLC-ESI/Q-TOF-MS/MS). The detailed procedures and results of the chemical composition analysis of alkaloids from RSH are provided in the Supplementary Materials (Figure S2 and Table S8).

#### 2.3.2. Collection and Screening of Targets and Construction of Network Diagrams

The TCMSP database was utilized to query the target proteins corresponding to the alkaloid components. And the corresponding gene names were obtained by protein database (UniProt) (https://www.uniprot.org/, accessed on 12 July 2023) correction. The PubChem database (https://pubchem.ncbi.nlm.nih.gov/, accessed on 12 July 2023) was used to obtain the SDF files of the 2D structures of the compounds and imported into the database of the Swiss Target Prediction platform (http://www.swisstargetprediction.ch/, accessed on 12 July 2023). The target proteins of the compositions were obtained after removing the duplicated targets. Using “rheumatoid arthritis” as the keyword, the GeneCards (https://www.genecards.org/, accessed on 12 July 2023) database was used to search for relevant RA disease targets. A Venn diagram of the active compound’s target of action versus the RA disease target was plotted by the Venny 2.1 (https://bioinfogp.cnb.csic.es/tools/venny/index.html, accessed on 12 July 2023) online tool, and the target at the intersection of the two was taken as the potential target of ARSHs acting on RA. The RA potential targets (intersecting targets) were imported into the STRING platform (https://cn.string-db.org/, accessed on 12 July 2023), the protein species was set to “homo sapiens”, and the minimum interaction threshold was set to medium “medium confidence” (0) and “Confidence” (0.9). The protein interaction relationship was obtained, and the TSV file was downloaded and imported into Cytoscape 3.8.2 software to construct the PPI network diagram which was analyzed by the ‘Network Analyzer’ in the software. The nodes with degree, betweenness centrality, and closeness centrality values greater than the average were identified as the key targets of ARSHs against RA. The components corresponding to the intersecting targets were mapped out by Excel and imported into Cytoscape 3.8.2 software to construct a network diagram of “active ingredient-intersecting targets”. The key components of ARSHs against RA were screened out with the help of “Network Analyzer” as described above.

#### 2.3.3. GO Analysis and KEGG Pathway Enrichment Analysis

After converting the key core targets into corresponding target IDs via the g: Profiler website (https://galaxy.hpc.ut.ee/, accessed on 12 July 2023), GO enrichment analysis and KEGG signaling pathway analysis were performed using the OmicShare cloud platform.

#### 2.3.4. Molecular Docking of Components and Targets

The 2D structures of the main key components were obtained from the PubChem database (https://pubchem.ncbi.nlm.nih.gov/, accessed on 15 July 2023), and the 3D structures were saved in mol2 format after importing them into ChemBio3D. The protein crystal complexes of the key targets were searched in the PDB database (https://www.rcsb.org/, accessed on 15 July 2023), and the target proteins were separated from the original ligands by removing water molecules, phosphates, and excess inactive ligands in the proteins with the help of PyMOL 2.5.6 software. The separated target proteins were imported into AutoDock Tools 4.2.6 softwarefor hydrogenation and charge addition. The target proteins, docking components, and proto-ligands were then converted to pdbqt format in AutoDock Tools 4.2.6 software. Molecular docking was performed using the site where the proto-ligand was located as the active pocket to verify the affinity between the component and the target protein.

### 2.4. Validation Experiments on the Anti-RA Effects and Mechanisms of ARSHs

#### 2.4.1. Proliferation Inhibition of Key ARSHs on MH7A Cells

MH7A cells in the logarithmic growth phase were adjusted to 5 × 10^3^ cells/mL and inoculated in 96-well plates (100 µL/well). After the cells were attached to the wall, the treatment groups were added with gradient concentrations (1000~7.8125 µg/mL) of dihydrochelerythrine, magnoflorine, and nitidine chloride, respectively, and there were negative control and blank groups (*n* = 6).

MTT assay: MH7A cells in the logarithmic growth phase were harvested, and the cell concentration was adjusted to a single-cell suspension of 5 × 10^3^ cells/mL. The suspension was seeded into 96-well plates at 100 µL per well. After the cells adhered and grew for 24 h, the administration groups were treated with different concentrations of dihydrochelerythrine, magnoflorine, and nitidine chloride (final concentrations: 1000, 500, 250, 125, 62.5, 31.25, 15.625, and 7.8125 µg/mL), respectively. Meanwhile, a negative control group and a blank group were set up, with six replicate wells in each group. After further incubation for 24 h, the culture medium was discarded. Then, 20 µL of 5 mg/mL MTT was added to each well, followed by incubation in the dark for 4 h. Subsequently, 150 µL of DMSO was added to each well to dissolve the formed formazan crystals. After shaking for 10 min to mix thoroughly, the OD value at 570 nm was measured to calculate the proliferation rate and IC50 according to Equation (1), and the experiment was repeated independently three times.

(1) $$\mathrm{Cell}\;\mathrm{proliferation}\;\mathrm{inhibition}\;(\%)=1-\frac{{OD}_{dministered\;group}-{OD}_{blank\;group}}{{OD}_{Negative\;group}-{OD}_{blank\;group}}\;\times 100\%$$

#### 2.4.2. Detection of Inflammatory Factors in MH7A Cells by ELISA

MH7A cells in the logarithmic growth phase were adjusted to 5 × 10^4^ cells/mL and inoculated in 24-well plates (500 µL/well). After incubation for 24 h until the cells were adhered to the wall, the medium containing dihydrochelerythrine, magnoflorine, nitidine chloride (final concentrations of 1/2, 1/4, and 1/8 of IC50), and MTX (final concentration of 116.8750 µg/mL) was added. Then TNF-α (final concentration of 20 ng/mL) was added to intervene for 24 h. The cell supernatants were collected to determine the levels of IL-17A, IL-6, and IL-1β according to the kit instructions.

#### 2.4.3. Determination of Apoptosis in MH7A Cells by Flow Cytometry

MH7A cells in the logarithmic growth phase were adjusted to 2 × 10^6^ cells/mL and inoculated in 6-well plates (2 mL/well). After incubation for 24 h until the cells were adhered to the wall, the treatment group was supplemented with dihydrochelerythrine, magnoflorine, nitidine chloride (final concentration of 1/8 IC50 + TNF-α), and MTX (116.8750 µg/mL + TNF-α), respectively, while the blank group had no treatment and the model group was supplemented with TNF-α only. The cells were cultured for 48 h after drug administration; the medium was discarded, washed with PBS, and collected by trypsin digestion and centrifugation. The blank group was incubated with Apoptosis Positive Control Solution in an ice bath for 30 min. The cells in each group were washed with PBS and resuspended in Binding Buffer, and then stained with Annexin V-FITC/PI for 15 min. The apoptosis rate was detected by flow cytometry.

#### 2.4.4. Western Blot Detection of Bax, Bcl-2, SRC, STAT3, MAPK3 Expression

MH7A cells were treated according to 2.4.3, washed with PBS, and lysate was added to a lysis solution for ice bath lysis for 30 min (intermittent blowing). The solution was centrifuged at 12,000 rpm at 4 °C for 10 min, and the supernatant was taken to quantify the total protein. Separation gel (TEMED to promote coagulation) and 5% concentrated gel were sequentially perfused in the gel maker, and the comb was inserted. After gelation, samples were taken, and electrophoresis was performed with electrophoresis solution at constant pressure. The PVDF membrane was activated by methanol for 2 min, and the membrane transfer folder was assembled with the gel, filter paper, and sponge, and the membrane was flowed at a constant flow rate of 300 mA for 30 min (cooled down by an ice bath). The membrane was rinsed with TBST and closed with 5% skimmed milk powder for 30 min. Primary antibody was incubated at 4 °C overnight and washed with TBST 3 times (5 min/time); HRP-labeled secondary antibody (1:5000 TBST) was incubated at room temperature for 30 min, and the membrane was washed in the same way; ECL luminescent solution (A/B solution 1:1) covered the membrane surface uniformly and then reacted for 1 min before analysis by the chemiluminescence imaging instrument.

### 2.5. Statistical Analysis

Prior to statistical analysis, we conducted tests for normality. For data that met the normality assumption, we used *t*-tests and ANOVA; for data that did not meet the normality assumption, we used non-parametric tests. All statistical analyses were performed using SPSS 24.0 software. The data obtained from the experiment were initially organized using Excel 2019. GraphPad Prism 7.0 software was used for drawing the pictures. The results were expressed as mean ± standard deviation. SPSS 24.0 software was used to compare the differences between the groups, a *t*-test was used to compare the samples of two groups, and one-way ANOVA was used to compare the means of multiple samples. The statistical results *p* < 0.05 and *p* < 0.01 indicated that the differences were statistically significant.

## 3. Results

### 3.1. Activity of Different Extracts from RSH Against RA

#### 3.1.1. Effect of Different RSH Extracts on Arthritis Index and Toe Swelling in RA Rats

**Figure 1:**
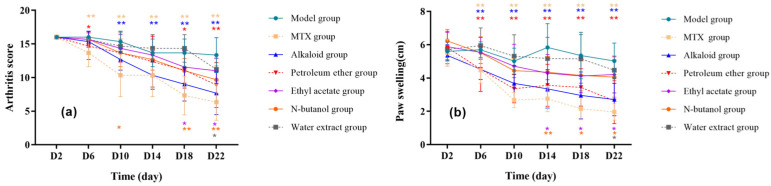
Effects of different extracts from RSH on (**a**) arthritis score and (**b**) toe swelling in RA rats. Note: Compared with the model group, * *p* < 0.05, ** *p* < 0.01.

As shown in Figure 1a, the effects of different extracts from RSH on the arthritis scores in RA rats showed dose-time differences. The MTX group significantly suppressed the arthritis scores on the 6th day after intervention (D6) (*p* < 0.05), the alkaloid group significantly reduced the scores from D10 onwards compared to the model group, the petroleum ether/ethyl acetate/n-butanol group showed anti-inflammatory effects at D18, and the water extract group did not show any improvement until D22. The arthritis index of each extraction site was ranked as follows: alkaloid group < petroleum ether group < n-butanol group < ethyl acetate group < water extract group, suggesting that alkaloid and petroleum ether from RSH have better anti-RA effects. On the other hand, complete Freund’s adjuvant induced redness and swelling of the right hind toe in rats, which peaked at D3–4, accompanied by joint enlargement and the limitation of movement. After drug treatment, swelling from D6 was significantly lower in the MTX, alkaloid, and petroleum ether groups than in the model group (*p* < 0.05); at D14 the treatment was effective in the ethyl acetate and n-butanol groups; and at D22 swelling was also improved in the water extract group (*p* < 0.05). The degree of swelling in each group, from low to high, was petroleum ether group < alkaloid group < n-butanol group < water extract group < ethyl acetate group, suggesting that the petroleum ether and alkaloid extracts had the best anti-inflammatory effects, which could significantly alleviate the inflammation of the joints (Figure 1b).

#### 3.1.2. Effect of Different RSH Extracts on Inflammatory Factors in RA Rat Serum

**Figure 2:**
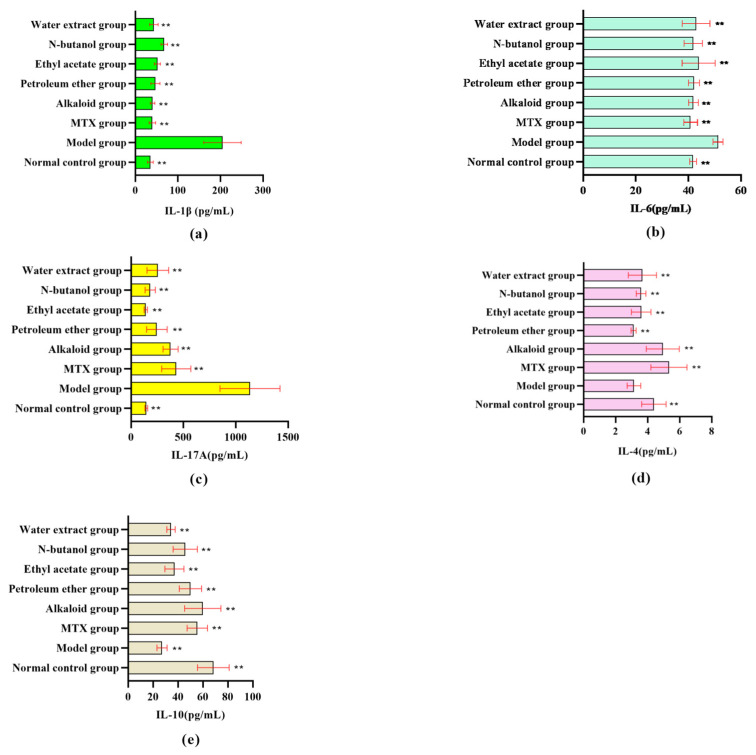
Effect of different extracts from RSH on inflammatory factors in sera of RA rats. (**a**) IL-1β, (**b**) IL-6, (**c**) IL-17A, (**d**) IL-4, and (**e**) IL-10. Note: Compared with the model group, ** *p* < 0.01.

The serum levels of IL-1β, IL-6, and IL-17A were significantly higher in the model group than in the blank group (*p* < 0.05). After administration, the levels of the above inflammatory factors in different RSH extracts and MTX groups were significantly lower than those in the model group (*p* < 0.05). Among them, the levels of IL-1β in the alkaloid group and water extract group (Figure 2a), IL-6 in the n-butanol group and alkaloid group (Figure 2b), and IL-17A in the ethyl acetate group and n-butanol group (Figure 2c) were relatively lower. It is suggested that different extracts from RSH can alleviate the inflammatory response of RA by inhibiting the expression of pro-inflammatory factors.

On the other hand, for the levels of anti-inflammatory factors, the levels of IL-4/IL-10 in serum were significantly lower in the model group compared to the blank group (*p* < 0.05). After the drug intervention, the IL-4 level in the alkaloid group was significantly rebounded (Figure 2d), and all the extracts were able to up-regulate the expression of IL-10 (Figure 2e), in which the restored level of IL-4/IL-10 in the alkaloid group was comparable to that of the positive control group. This result suggests that the extracts from RSH regulate Th1/Th2 immune balance by up-regulating the expression of anti-inflammatory factors (IL-4/IL-10), in which the activation of IL-4 was more significant in the alkaloid and n-butanol extracts.

### 3.2. Network Pharmacological Analysis of Alkaloids from RSH (ARSHs) Against RA

#### 3.2.1. Confirmation of the Composition of ARSHs

In vivo activity screening of different extracts indicated that the ARSHs exhibited better anti-RA effects. Therefore, ARSHs were selected for further study to investigate the possible anti-RA mechanism of RSH. Among the results of UPLC-ESI/Q-TOF-MS/MS compositional analysis and blood-entering components analysis, the compounds with well-defined structures were retained among the active compounds obtained from the preliminary screening (for the next step of target prediction) and were numbered according to the molecular numbering in the TCMSP database.

#### 3.2.2. Network Pharmacologic Analysis

**Figure 3:**
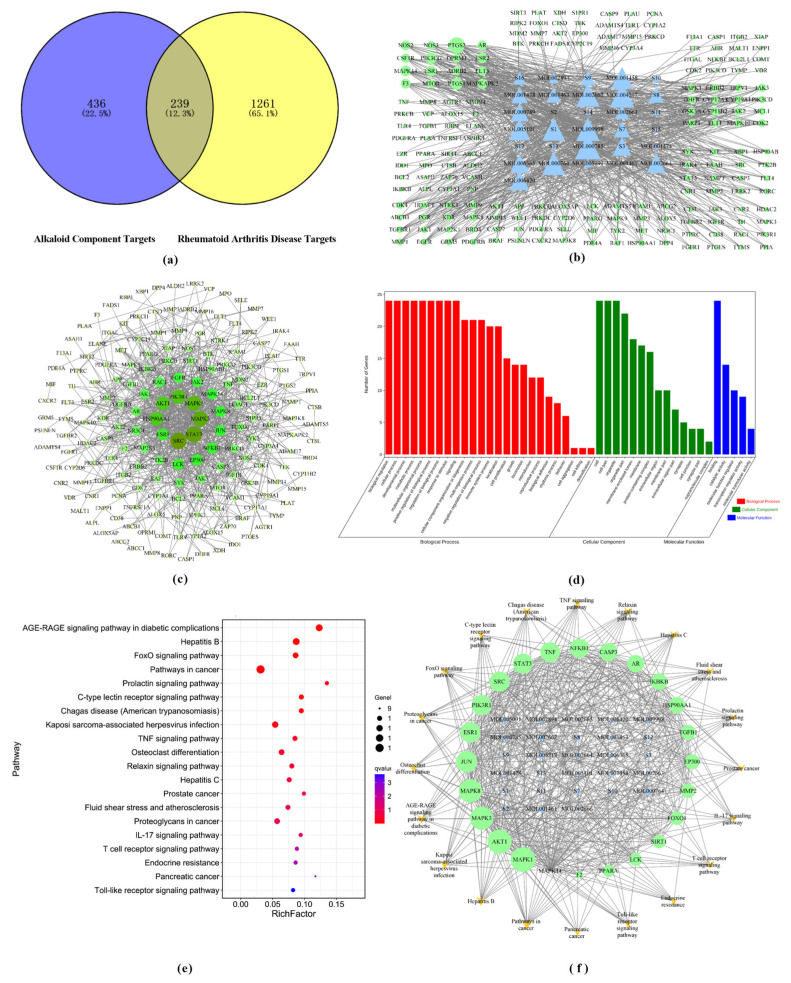
Network pharmacologic analysis of ARSHs against RA. (**a**) Wayne diagram of ARSHs’ active compound–disease intersecting genes. (**b**) ARSHs’ “compound–target protein” network. (**c**) PPI network diagram of intersecting targets of ARSHs for RA; (**d**) GO entries of key targets; (**e**) bubble diagram of pathway enrichment analysis; (**f**) “component–target–pathway” network of ARSHs against RA.

A total of 675 targets related to alkaloidal active ingredients were collected from TCMSP and Swiss Target Prediction databases after removing duplicates. The RA-related targets were retrieved from the GeneCards database, and the top 1500 RA disease targets were selected according to the correlation score results in descending order. Then 239 intersection targets of RA disease targets and alkaloidal component targets were identified as potential ARSH anti-RA targets (Figure 3a). The components and intersecting targets were imported into Cytoscape 3.8.2 to construct the component–target network, which contained 31 compounds, 187 targets, and 848 edges. After topological analysis, 15 compounds (e.g., dihydrochelerythrine, liriodenine, etc.) were higher than the average value of degree, median, and proximity to the center, which were the key components of anti-RA; among the disease targets, 55 targets had higher than average values of the corresponding parameter, and the degree values of PTGS2, PTGS1, etc., were the highest, reflecting the multi-component and multi-target characteristics of ARSHs (Figure 3b). The STRING database was utilized to construct a PPI network (human protein, confidence level ≥ 0.9) for anti-RA ARSHs, containing 183 nodes and 785 edges (4 isolated targets were not shown). Network topology analysis screened 24 key targets (degree value, median, and proximity centrality were higher than the mean), with SRC (50), STAT3 (48), MAPK3 (45), and MAPK1 (43) having the highest degree values. These targets were highly associated with other targets and may have been the core targets of ARSHs’ anti-RA capabilities (Figure 3c). GO enrichment analysis of 24 key targets was performed by the OmicShare platform, and 2072 entries were screened (*p* < 0.01 and FDR < 0.05). Biological process (BP) was enriched with 1842 entries, involving lipid response, DNA-binding transcription factor regulation, intracellular signaling, etc. Cellular component (CC) contained 83 entries, involving membrane rafts, membrane microregions, nucleoplasm, etc., and molecular function (MF) had 147 entries, including enzyme binding, kinase activity, transcription factor binding, etc. The results suggest that ARSHs may exert anti-RA effects through multiple pathways regulating biological processes (Figure 3d). Pathway enrichment analysis revealed that 24 key targets were significantly enriched in 128 pathways (*p* < 0.01, FDR < 0.01). The top 20 pathways involved the AGE-RAGE signaling pathway in diabetic complications, TNF signaling pathway, osteoclast differentiation, IL-17 signaling pathway, T cell receptor signaling pathway, Toll-like receptor signaling pathway, and so on, suggested that ARSHs act on multiple pathways to treat RA (Figure 3e). The key targets were mapped to their corresponding components and pathways, which were imported into Cytoscape 3.8.2 softwareto construct a “Component–Key Target–Pathway” visualization network. The network included 72 nodes (28 compounds, 24 targets, and 20 pathways) and 554 edges. The results suggested that the active components of ARSHs were distributed in different pathways and synergistically exerted their therapeutic effects on RA, and the key targets were also distributed in different pathways, indicating that the enriched RA pathway could be an important pathway for ARSHs against RA (Figure 3f).

### 3.3. Molecular Docking Analysis

**Figure 4:**
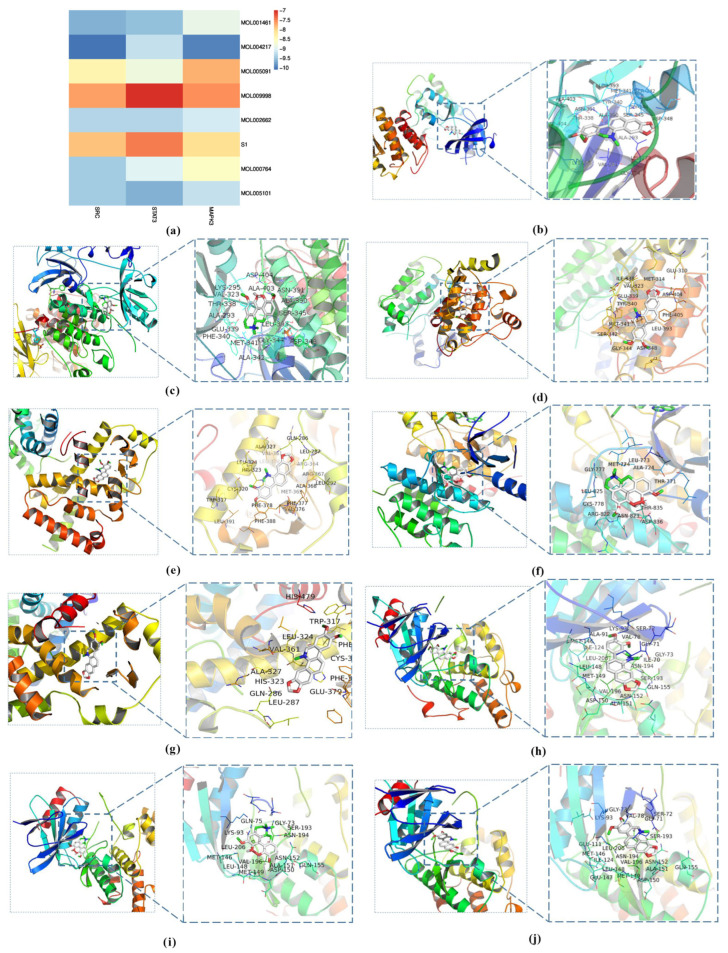
Molecular docking analysis of ARSHs against RA. (**a**) Heat map of molecular docking results; (**b**) dihydrochelerythrine docked with SRC receptor; (**c**) magnoflorine docked with SRC receptor; (**d**) nitidine chloride docked with SRC receptor; (**e**) dihydrochelerythrine docked with STAT3 receptor; (**f**) magnoflorine docked with STAT3 receptor; (**g**) nitidine chloride docked with STAT3 receptor; (**h**) dihydrochelerythrine docked with MAPK3 receptor docking; (**i**) magnoflorine docking with MAPK3 receptor; (**j**) nitidine chloride docking with MAPK3 receptor.

Molecular docking of seven ARSH components (e.g., dihydrochelerythrine, liriodenine, etc.) with the key targets SRC, STAT3, and MAPK3 was performed based on the network pharmacology screening (Figure 4a). The results showed that the binding energies of all the components to the targets ranged from −7.0 to −10.1 kcal/mol (binding energies ≤ −7.0 kcal/mol suggesting strong binding activity), among which dihydrochelerythrine, magnoflorine, and nitidine chloride had the best affinities to SRC, STAT3, and MAPK3. PyMOL 2.5.6 software visualization indicated that these components could be stably embedded in the active pocket of target proteins, which validated the reliability of the predicted results (Figure 4b–j).

### 3.4. Experimental Validation of the Predicted Targets of ARSHs Against RA

#### 3.4.1. Effect of ARSH Components on the Viability of MH7A Cells

**Figure 5:**
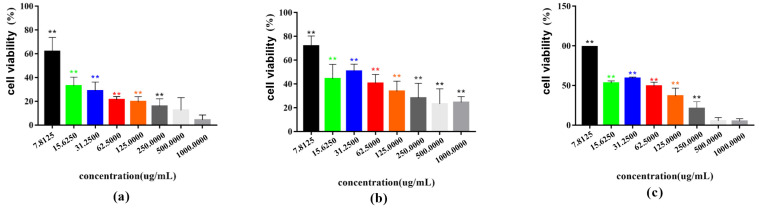
Effect of ARSH components on the viability of MH7A cells. (**a**) Magnoflorine group, (**b**) nitidine chloride group, and (**c**) dihydrochelerythrine group. ** *p* < 0.01.

**Figure 6:**
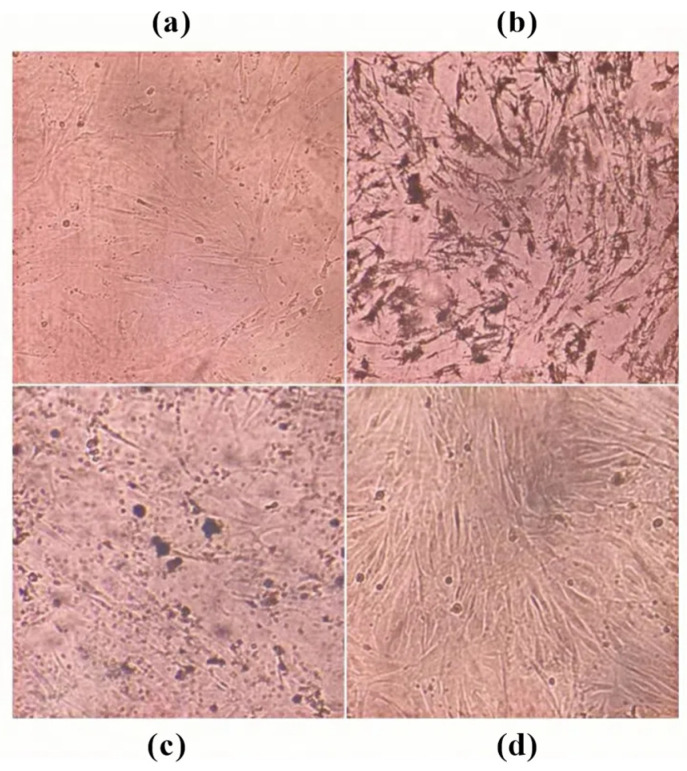
Morphology of MH7A cells after intervention with ARSH components. (**a**) Magnoflorine group; (**b**) nitidine chloride group; (**c**) dihydrochelerythrine group; (**d**) blank group. All groups at a drug concentration of 62.50 µg/mL.

Magnoflorine, nitidine chloride, and dihydrochelerythrine inhibited the viability of MH7A cells concentration-dependently (all *p* < 0.05). Magnoflorine (IC50 = 789.65 ± 22.66 µg/mL) and dihydrochelerythrine (IC50 = 360.35 ± 0.95 µg/mL) were effective at concentrations ranging from 31.25 to 1000 µg/mL. Nitidine chloride (IC50 = 481.22 ± 7.03 µg/mL) was effective at concentrations ranging from 7.81 to 1000 µg/mL (Table 2) (Figure 5). Inverted microscopic observation showed that cells were attenuated and growth density was reduced after the intervention of each monomer (Figure 6). Based on the ordering of IC50 values (magnoflorine > nitidine chloride > dihydrochelerythrine), one-half, one-quarter, and one-eighth concentration gradients of IC50 were selected for validation in subsequent experiments.

#### 3.4.2. Effect of ARSH Components on Inflammatory Factors in MH7A Cells

**Figure 7:**
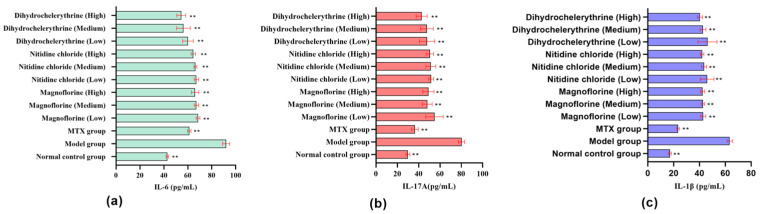
Effect of ARSHs on inflammatory factor levels in MH7A cells. (**a**) IL-6 level; (**b**) IL-17A level; (**c**) IL-1β level. ** *p* < 0.01.

The results showed that under the stimulation of inflammatory factor TNF-α, the contents of IL-6, IL-17A, and IL-1β were increased in MH7A cells of the model group compared to those of the blank group (*p* < 0.01) (Figure 7). After administration, the contents of IL-6, IL-17A, and IL-1β in MH7A cells in different concentrations of the magnoflorine, nitidine chloride, and dihydrochelerythrine groups were reduced compared to those in the model group (*p* < 0.01). The above results suggest that the ARSH components magnoflorine, nitidine chloride, and dihydrochelerythrine can effectively reduce the TNF-α-induced secretion of the inflammatory factors IL-6, IL-17A, and IL-1β by MH7A cells, exhibiting favorable anti-RA activity.

#### 3.4.3. Effect of ARSH Components on Apoptosis in MH7A Cells

**Figure 8:**
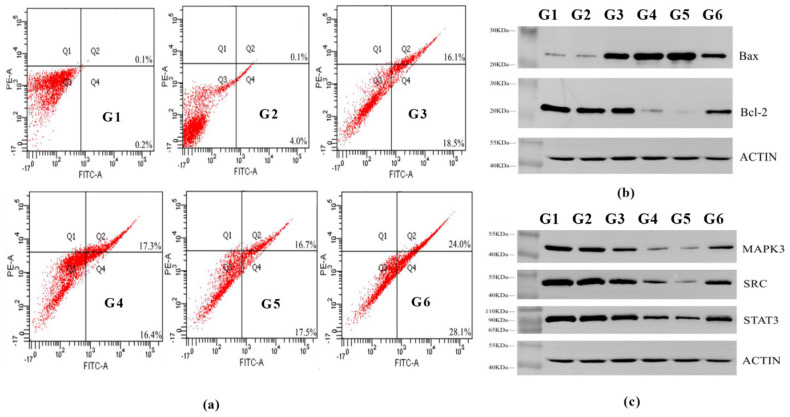
Effect of ARSH components on apoptosis in MH7A cells. (**a**) Effects of the main components of ARSHs on the apoptosis of MH7A cells. G1: blank group, G2: model group, G3: magnoflorine group, G4: nitidine chloride group, G5: dihydrochelerythrine group, and G6: MTX group. (**b**) Apoptosis-related proteins detected by WB. G1: model group, G2: blank group, G3: MTX group, G4: nitidine chloride group, G5: dihydrochel-erythrine group, and G6: magnoflorine group. (**c**) MAPK3, SRC, and STAT3 protein expression in MH7A cells detected by WB. G1: model group, G2: blank group, G3: MTX group, G4: nitidine chloride group, G5: dihydrochelerythrine group, and G6: magnoflorine group. And the concentrations are all one-eighth of their IC50.

Magnoflorine, nitidine chloride, and dihydrochelerythrine significantly induced apoptosis in MH7A cells (apoptosis rates of 35.6%, 34.7%, and 34.2%, respectively; all *p* < 0.01), which was consistent with the trend of the positive drug MTX (52.1%), as shown in Figure 8a, suggesting that they inhibited the cell proliferation through exerting pro-apoptosis and anti-RA effects. Western blot analysis showed that the above components could down-regulate the expression of the anti-apoptotic factor Bcl-2 and up-regulate the expression of the pro-apoptotic factor Bax, suggesting that they promoted apoptosis by regulating the Bcl-2 and Bax pathways (Figure 8b). In addition, compared to the blank and model groups, magnoflorine, nitidine chloride, dihydrochelerythrine, and MTX were able to reduce the expression of SRC, STAT3, and MAPK3 proteins after the intervention of MH7A cells (Figure 8c). This also verified the results of network pharmacology and molecular docking which found that magnoflorine, nitidine chloride, and dihydrochelerythrine have a certain affinity for SRC, STAT3, and MAPK3 and are able to exert anti-RA effects by down-regulating the expression of SRC, STAT3, and MAPK3 proteins.

## 4. Discussion

According to the literature [18,19], we subcutaneously injected CFA into the foot-plantar of rats to establish an RA rat model. After injection, the rats showed redness and swelling of the forefoot or hindfoot joints, obvious swelling of the ankle joints, and dragging phenomena in severe cases, which were consistent with those reported in the literature [20], and this was regarded as a successful modeling. Subsequent experiments showed that ARSHs significantly reduced foot-plantar swelling and the arthritis index scores of RA rats induced by complete Freund’s adjuvant (*p* < 0.01) and improved the pathological damage of the joints, which was superior to other polar extraction sites. The alkaloids showed the highest extract yield, suggesting that they were the main active components of RSH. It has been demonstrated that alkaloids exert anti-RA effects through multi-targeted actions (inhibition of inflammatory factors, modulation of the JNK/NF-κB pathway, and induction of apoptosis) [21,22]. In vitro experiments have shown that RSH can inhibit LPS-induced NO production in RAW 264.7 cells, but there is still a lack of research on its anti-RA active components and mechanisms. Therefore, the targets and pathways of ARSHs need to be further analyzed to provide a basis for anti-RA studies.

Pro-inflammatory factors such as IL-1β, IL-6, and IL-17A and anti-inflammatory factors such as IL-4 and IL-10 play a key role in the pathologic process of RA. Serum levels of pro-inflammatory factors (IL-1β, IL-6, IL-17A) and anti-inflammatory factors (IL-4, IL-10) were significantly elevated in RA rats in the model group, which were correlated with the progression of inflammation and bone destruction in RA [23,24,25]. Different extracts from RSH significantly reduced the levels of pro-inflammatory factors and elevated anti-inflammatory factors: the alkaloids had an IL-1β comparable to that of the positive drug and significantly elevated IL-4 (*p* < 0.05); the alkaloid and n-butanol extracts were superior for IL-6 inhibition; and the ethyl acetate group inhibited IL-17A most significantly. The anti-inflammatory factor IL-10 was significantly elevated in all extract groups, with the best effect in the alkaloid group. The results showed that ARSHs exerted anti-RA effects through bidirectional regulation of pro-inflammatory/anti-inflammatory factor balance (inhibition of IL-1β/IL-6/IL-17A and promotion of IL-4/IL-10), and their mechanisms may be related to the regulation of NF-κB, JAK/STAT, and other pathways [26,27,28], further validating alkaloids as the core active component of RSH.

Network pharmacological analysis predicted the components and mechanisms of ARSHs against RA, screening 15 key components (e.g., dihydrochelerythrine, magnoflorine, etc.) and 24 core targets (e.g., SRC, STAT3, MAPK3, etc.). SRC participates in cell growth and inflammatory signaling pathways as a tyrosine kinase [29]; STAT3 regulates immune response and inflammatory factor release [30,31]; and MAPK3 mediates the production of inflammatory factors and plays a key role in the downstream signaling cascade of IL-1/IL-17 [32,33,34]. The above targets are closely related to the inflammatory process of RA, suggesting that ARSHs can play a therapeutic role by modulating immune-inflammation-related pathways.

The 24 key ARSH anti-RA targets enriched by GO were mainly involved in the regulation of DNA-binding transcription factors, intracellular signaling (BP), membrane raft/membrane microcellular localization (CC), and transcription factor/kinase binding (MF). TNF-α exacerbates RA inflammation by inducing pro-inflammatory mediators; osteoclast differentiation factors promote bone resorption and IL-6/IL-1 secretion; and the IL-17 pathway activates transcription factors, such as NF-κB, which synergize with other pathways to exacerbate joint damage [35,36,37,38,39,40]. The above pathways are closely related to RA inflammation and bone destruction, suggesting that ARSH may exert anti-RA effects through the synergistic regulation of multiple pathways.

Molecular docking of seven ARSH components (dihydrochelerythrine, magnoflorine, etc.) with core targets (SRC, STAT3, MAPK3) based on network pharmacological screening showed that dihydrochelerythrine, magnoflorine, and nitidine chloride possessed strong binding activities (affinity < −7.0 kcal/mol), corroborating their multi-component–multi-target synergistic anti-RA potential. Further validation using MH7A cells as a model revealed that the above components significantly inhibited cell proliferation and reduced the secretion of TNF-α-induced pro-inflammatory factors IL-6, IL-17A, and IL-1β, with a trend of action consistent with that of the total ARSHs, suggesting that the single components exerted their anti-RA effects by inhibiting synovial fibroblast activation and inflammatory factor release.

The imbalance of synovial cell apoptosis (abnormal Bcl-2/Bax expression) in RA is a key mechanism leading to cartilage destruction [41,42,43]. Flow cytometry showed that magnoflorine, nitidine chloride, and dihydrochelerythrine significantly promoted apoptosis in MH7A cells (*p* < 0.01), and the mechanism was associated with the down-regulation of the anti-apoptotic protein Bcl-2 and up-regulation of the pro-apoptotic protein Bax. Western blot confirmed that the three could down-regulate the expression of SRC, STAT3, and MAPK3 proteins, which are the core targets predicted by network pharmacology, and verified the molecular mechanism that ARSHs exerted anti-RA effects by regulating apoptosis-related targets and signaling pathways.

The limitations of this study include the absence of Western blot analyses for phosphorylated forms of SRC, STAT3, and MAPK3, as well as molecular dynamics simulations. Due to time and resource constraints, these experiments were not conducted. Future studies will consider incorporating these analyses to further validate our findings. In addition, we plan to use HPLC-UV in future studies to provide absolute quantitative data on each alkaloid in the in vivo extract, thereby further verifying that their in vivo levels are sufficient to mediate the observed anti-rheumatoid arthritis effects.

The comprehensive results showed that ARSHs could synergistically regulate apoptosis, inflammatory factor secretion, and key signaling pathways to exhibit multidimensional anti-RA effects, which could provide a theoretical basis for their application.

## 5. Conclusions and Outlooks

Based on network pharmacology, molecular docking, and in vitro experimental validation, this study systematically revealed the multi-pathway action characteristics of the ARSHs against RA. In vivo animal experiments indicated that ARSHs possessed significant anti-RA activity. A total of 6 mg/kg/d for 22 days improved foot-plantar swelling and arthritis scores in RA rats and regulated the balance of pro-inflammatory factors, such as IL-1β, IL-6, and IL-17A, and anti-inflammatory factors, such as IL-4 and IL-10. Network pharmacology screened 15 key alkaloid components (e.g., dihydrochelerythrine, magnoflorine, etc.) and 24 core targets (e.g., SRC, STAT3, MAPK3) and confirmed their strong binding activities by molecular docking, suggesting that ARSHs can exert anti-RA effects by modulating immune-inflammation-related pathways. In vitro experiments further demonstrated that the above components significantly inhibited the proliferation of MH7A cells and induced apoptosis, and the mechanism was closely related to the down-regulation of the anti-apoptotic protein Bcl-2, the up-regulation of the pro-apoptotic protein Bax, and the inhibition of SRC/STAT3/MAPK3 expression. In addition, ARSHs can bidirectionally regulate the balance of pro-inflammatory/anti-inflammatory factors (inhibiting IL-1β/IL-6/IL-17A and elevating IL-4/IL-10), further alleviating joint inflammation and bone destruction. In the future, we can expand the study of the chemical composition and pharmacodynamics of polar sites of RSH, such as petroleum ether and ethyl acetate to achieve increased validation of the in vivo effects of active ingredients. Existing studies have initially revealed the anti-inflammatory and apoptosis regulatory pathways, but the RA-related signaling network and multi-target synergistic mechanisms still need to be systematically elucidated. In addition, the development of non-traditional medicinal parts such as the branches and leaves of RSH is recommended to enhance the resource utilization of RSH.

## Figures and Tables

**Table 1:** Arthritis index scoring criteria.

Characteristics	Score
Normal, no symptoms	0
Slight swelling or little erythema in ankle or wrist joints	1
Mild swelling and little erythema at ankle and wrist joints	2
Moderate swelling and more erythema at ankle to metatarsal or metacarpal joints	3
Severe swelling and a lot of redness from the ankle to the metatarsals	4

**Table 2:** IC50 of ARSH components on MH7A cells.

Groups	IC50 (μg/mL)
Magnoflorine	789.6492 ± 22.6635
Nitidine chloride	481.2247 ± 7.0282
Dihydrochelerythrine	360.3517 ± 0.9453

## Data Availability

The data presented in this study are available on request from the corresponding author. (The data are not publicly available due to privacy or ethical restrictions).

## Supplementary Materials

The following supporting information can be downloaded at: https://www.mdpi.com/article/10.3390/cimb47080661/s1.

## Abbreviations

The following abbreviations are used in this manuscript:

Bax	Bcl-2-associated X protein
Bcl-2	B-cell lymphoma-2
BP	biological process
CC	cellular component
CFA	complete Freund’s adjuvant
CMC-Na	carboxymethyl cellulose-Na
DMARDs	disease-modifying antirheumatic drug
DMSO	dimethyl sulfoxide
ELISA	enzyme-linked immunosorbent assay
GCs	glucocorticosteroid
GO	gene ontology
IC_50_	half maximal inhibitory concentration
IL	interleukin
KEGG	Kyoto Encyclopedia of Genes and Genomes
MAPK1	mitogen-activated protein kinase 1
MAPK3	mitogen-activated protein kinase 3
MF	molecular function
MH7A	fibroblast-like synovial cells of human rheumatoid arthritis
MTT	thiazolyl blue tetrazolium bromide
MTX	methotrexate
NASIDs	nonsteroidal anti-inflammatory drugs
OD	optical density
One-way ANOVA	one-way analysis of variance
PBS	phosphate-buffered saline
PMSF	phenylmethanesulfonyl fluoride
PPI	protein–protein interaction
PVDF	polyvinylidene fluoride
RA	rheumatoid arthritis
RIPA	radioimmunoprecipitation assay buffer
SDS	sodium dodecyl sulfate
SRC	proto-oncogene tyrosine-protein kinase Src
STAT3	signal transducer and activator of transcription 3
TBST	Tris-HCl and Tween solution
TCMSP	Traditional Chinese Medicine Systems Pharmacology Database and Analysis Platform
TEMED	N,N,N′,N′-tetramethylethylenediamine
TNF-α	tumor necrosis factor-α
Tris	Tris hydroxyl methyl aminomethan
WB	Western blot
