# Supplementary material for: Potential of Alkaloids from Zanthoxylum nitidum var. tomentosum in Treating Rat Rheumatoid Arthritis Model and Validation of Molecular Mechanisms
Source: Curr Issues Mol Biol. 2025 Aug 15;47(8):661. doi: 10.3390/cimb47080661 (PMC12384453; doi:10.3390/cimb47080661)
Supplement: Supplementary file 1 [file cimb-47-00661-s001.zip › cimb-3743670-supplementary.pdf]

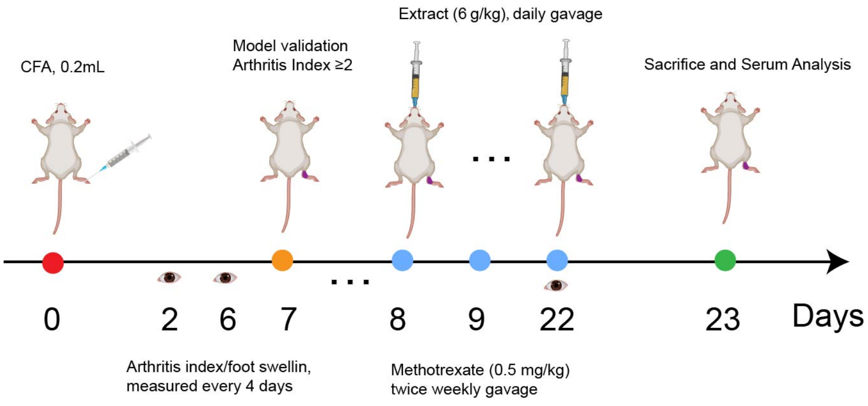

Figure S1. Protocol flow chart for drug administration in the RA rat model.

Experimental Reagents and Instruments

2.2 Experimental reagents and instruments used in the study are listed in Tables S1 and S2.

Table S1 Information of experimental reagents

Name	Manufacturer	Batch Number
Sodium carboxymethyl cellulose (CMC-Na)	Tianjin Damao Chemical Reagent Factory	20211004
Normal saline	Guangxi Yuyuan Pharmaceutical Co., Ltd.	H21102103
Methotrexate (MTX)	Shanghai SINE Pharmaceutical Co., Ltd.	036200804
Complete Freund's adjuvant (CFA)	Sigma-Aldrich, USA	F5881
Chloral hydrate	Chengdu Kelong Chemical Co., Ltd.	20200923021
Anhydrous ethanol	Xilong Scientific Co., Ltd.	2022042002
Rat IL-6 ELISA kit	Thermo Fisher Scientific	MAN0016898
Rat IL-1β ELISA kit	Thermo Fisher Scientific	MAN0016903
Rat IL-4 ELISA kit	Thermo Fisher Scientific	MAN0016900
Rat IL-10 ELISA kit	Thermo Fisher Scientific	MAN0016902
Rat IL-17A ELISA kit	Wuhan Huamei Biotechnology Co., Ltd.	CSB-E07451r

Table S2 Information of experimental instruments

Name	Manufacturer
JJ500 electronic balance	Changshu Shuangjie Testing Instrument Factory
ST16R low-temperature centrifuge	Thermo Fisher Scientific
SL01-1 vernier caliper	Deqing Shengqinxin Electronics Technology Co., Ltd.
BX43 upright optical microscope	Olympus Corporation
MSX2 imaging system	Guangzhou Mingmei Optoelectronics Technology Co., Ltd.
Forma 8900 ultra-low temperature freezer	Thermo Fisher Scientific
ST16R low-temperature centrifuge	Thermo Fisher Scientific
Multiskan Sky full-wavelength microplate reader	Thermo Fisher Scientific
50–300 μL multichannel pipette	Dragon Lab
20–200 μL pipette	Thermo Fisher Scientific
50–1000 μL pipette	Thermo Fisher Scientific

2.3 The experimental reagents and instruments used in the study are listed in Tables S3, S4, and S5.

Table S3 Chemical Reference Substances

Name	Batch Number	Manufacturer	CAS NO
Nitidine chloride (purity 99.81%)	MUST-22111707	Chengdu Mansite Biotechnology Co., Ltd.	13063-04-2
Dihydrochelerythrine (purity 99.21%)	MUST-22032004	Chengdu Mansite Biotechnology Co., Ltd.	6880-91-7
Chelerythrine hydrochloride (purity 99.41%)	MUST-21101002	Chengdu Mansite Biotechnology Co., Ltd.	34316-15-9
Sanguinarine hydrochloride (purity 99.50%)	MUST-23022212	Chengdu Mansite Biotechnology Co., Ltd.	5578-73-4
Zanthoxylin (purity 99.94%)	MUST-21101701	Chengdu Mansite Biotechnology Co., Ltd.	524-15-2
Woolly dictamnus (purity 99.91%)	MUST-22121906	Chengdu Mansite Biotechnology Co., Ltd.	483-90-9

Dictamnine (purity 99.98%)	MUST-22100807	Chengdu Mansite Biotechnology Co., Ltd.	484-29-7
Skimmianine (purity 99.62%)	PRF10081202	Chengdu Prifa Technology Development Co., Ltd.	83-95-4
Tetrahydropalmatine hydrochloride (purity 99.97%)	PRF21102042	Chengdu Prifa Technology Development Co., Ltd.	6024-83-5
Toddalolactone (purity 99.50%)	PRF20082821	Chengdu Prifa Technology Development Co., Ltd.	4335-12-0
Higenamine (purity 97.00%)	Y-115-150716	Chengdu Refines Biological Technology Co., Ltd.	5843-65-2
Palmatine hydrochloride (purity 98.75%)	MUST-21022604	Chengdu Mansite Biotechnology Co., Ltd.	10605-02-4
Jatrorrhizine hydrochloride (purity 99.50%)	MUST-23022212	Chengdu Mansite Biotechnology Co., Ltd.	960383-96-4
Magnoflorine (purity 99.82%)	MUST-22020816	Chengdu Mansite Biotechnology Co., Ltd.	7224-58-0

Table S4 Experimental Reagents

Reagent Name	Manufacturer	Cat. No.
Purified water (MS-grade)	Thermo Fisher Scientific	W6-4
Methanol (MS-grade)	Thermo Fisher Scientific	A456-4
Acetonitrile (MS-grade)	Thermo Fisher Scientific	A955-4
Formic acid (MS-grade)	Thermo Fisher Scientific	A117-50

Table S5 Experimental Instruments

Instrument Name	Manufacturer
UPLC-H CLASS ultra-performance liquid chromatography	Waters Corporation, USA
XEVO-G2 S qTOF quadrupole time-of-flight mass spectrometer	Waters Corporation, USA

Mix-3000 vortex mixer	Hangzhou Mio Instrument Co., Ltd.
Mikro 220R desktop high-speed refrigerated centrifuge	Hettich Scientific Instruments, Germany
Savant™ SPD131DDA SpeedVac™ vacuum centrifugal concentrator	Waters Corporation, USA
ACQUITY UPLC HSS T3 Column (1.7 μm, 2.1 mm×100 mm)	Waters Corporation, USA
LAB-300 manual multichannel pipette	Rainin, USA
LAB-1200 manual multichannel pipette	Rainin, USA
MassLynx mass spectrometry data acquisition software	Waters Corporation, USA

2.4 The experimental reagents and instruments used in the study are listed in Tables S6 and S7.

Table S6 Information of Drugs and Experimental Reagents

Name	Manufacturer	Batch Number
Magnoflorine (purity 99.02%)	Chengdu Mansite Biotechnology Co., Ltd.	MUST-21072708
Nitidine chloride (purity 91.00%)	National Institutes for Food and Drug Control	110848-201604
Dihydrochelerythrine (purity 99.21%)	Chengdu Mansite Biotechnology Co., Ltd.	MUST-22032004
Fetal bovine serum (FBS)	Cyagen Biosciences	T210420H501
DMEM medium	Gibco, USA	8122339
Penicillin-streptomycin mixture	Beijing Solarbio Science & Technology Co., Ltd.	20211120
0.25% Trypsin-EDTA solution	Gibco, USA	2376021
Phosphate-buffered saline (PBS)	Gibco, USA	8122098
Dimethyl sulfoxide (DMSO)	Beijing Solarbio Science & Technology Co., Ltd.	814O035
3-(4,5-Dimethylthiazol-2-yl)-2,5-diphenyltetrazolium bromide (MTT)	Shanghai Aladdin Biochemical Technology Co., Ltd.	917Q0514
Recombinant human TNF-α	PEPROTECH, USA	031825

Human IL-17A ELISA kit	Wuhan Elabscience Biotechnology Co., Ltd.	AK02B4FN8085, FU048Z866395
Human IL-6 ELISA kit	Wuhan Elabscience Biotechnology Co., Ltd.	AK04PINN9649, FU05XZ487490
Human IL-1β ELISA kit	Wuhan Elabscience Biotechnology Co., Ltd.	AK03002V8867, FU06HJV68585
Annexin V-FITC/PI apoptosis detection kit	Hangzhou Lianke Biotechnology Co., Ltd.	A20821
RIPA lysis buffer	Wuhan Servicebio Technology Co., Ltd.	CR2206025
Phosphatase inhibitor cocktail	Wuhan Servicebio Technology Co., Ltd.	CR2212012
Phenylmethanesulfonyl fluoride (PMSF)	Wuhan Servicebio Technology Co., Ltd.	CR2201056
Phosphorylation protease inhibitor	Wuhan Servicebio Technology Co., Ltd.	CR2210042
SDS-PAGE gel preparation kit	Wuhan Servicebio Technology Co., Ltd.	CR2211110
Protein Marker	Wuhan Servicebio Technology Co., Ltd.	MPC2211012
Polyvinylidene fluoride (PVDF) membrane	Wuhan Servicebio Technology Co., Ltd.	0000192418
Non-fat milk powder	Wuhan Servicebio Technology Co., Ltd.	CR2211051
Tween-20	Wuhan Servicebio Technology Co., Ltd.	CR2210018
Enhanced chemiluminescence (ECL) reagent	Wuhan Servicebio Technology Co., Ltd.	CR2211098-2
Ultra-sensitive ECL reagent	Wuhan Servicebio Technology Co., Ltd.	CR209031
Transfer buffer	Wuhan Servicebio Technology Co., Ltd.	20241008
Electrophoresis buffer	Wuhan Servicebio Technology Co., Ltd.	20241024
Tris-buffered saline (TBS)	Wuhan Servicebio Technology Co., Ltd.	20220208
Anti-SRC rabbit antibody	Wuhan Servicebio Technology Co., Ltd.	772620072902
Anti-STAT3 rabbit antibody	Wuhan Servicebio Technology Co., Ltd.	AC221013023
Anti-MAPK3 rabbit antibody	Wuhan Servicebio Technology Co., Ltd.	96917092605

Anti-BAX rabbit antibody	Wuhan Servicebio Technology Co., Ltd.	C103321062303
Anti-BCL-2 rabbit antibody	Wuhan Servicebio Technology Co., Ltd.	C82721042102
RP-conjugated goat anti-rabbit IgG	Wuhan Servicebio Technology Co., Ltd.	CR2210103
HRP-conjugated goat anti-mouse IgG	Wuhan Servicebio Technology Co., Ltd.	CR2209064

Table S7 Information of Experimental Instruments

Name	Manufacturer
Forma 8900 ultra-low temperature freezer	Thermo Fisher Scientific
ST16R low-temperature centrifuge	Thermo Fisher Scientific
Multiskan Sky full-wavelength microplate reader	Thermo Fisher Scientific
MLS-3781L autoclave	Panasonic Corporation, Japan
MCO-18AIC CO_2 incubator	Sanyo Electric Co., Ltd.
CK40 inverted microscope	Olympus Corporation
SW-CJ-1FD vertical clean bench	Suzhou Purification Equipment Co., Ltd.
MH-2 micro-oscillator	Qilinbeier Instrument Manufacturing Co., Ltd.
LSR Fortessa flow cytometer	BD Biosciences, USA
50–300 μL multichannel pipette	Dragon Lab
20–200 μL pipette	Thermo Fisher Scientific
50–1000 μL pipette	Thermo Fisher Scientific
KZ-III-F grinder	Wuhan Servicebio Technology Co., Ltd.
D3024R desktop high-speed refrigerated centrifuge	Dragon Lab
MS6000 palm centrifuge	Wuhan Servicebio Technology Co., Ltd.
MV-100 vortex mixer	Wuhan Servicebio Technology Co., Ltd.
MS-150 magnetic stirrer	Wuhan Servicebio Technology Co., Ltd.
DS-2S100 decolorizing shaker (pendulum type)	Wuhan Servicebio Technology Co., Ltd.
SVE-2 vertical electrophoresis system	Wuhan Servicebio Technology Co., Ltd.
SVT-2 transfer electrophoresis system	Wuhan Servicebio Technology Co., Ltd.
SPR80 ice maker	SIMAG
SPW-6S electrophoresis power supply	Wuhan Servicebio Technology Co., Ltd.
JY92-11N ultrasonic cell disruptor	Ningbo Xinzhi Biology

6100 chemiluminescence imager	Shanghai Qinxiang Scientific Instruments Co., Ltd.

Chemical Composition Analysis of Alkaloid from Rushanhu

1 Experimental Materials and Instruments

1.1 Materials and Reference Substances

The experimental material was the alkaloid fraction of Rushanhu. The reference substances used for determination included: nitidine chloride (99.81%), dihydrochelerythrine (99.21%), chelerythrine hydrochloride (99.41%), sanguinarine hydrochloride (99.50%), zanthoxylin (99.94%), woolly dictamnusin (99.91%), dictamnine (99.98%), palmatine hydrochloride (98.75%), jatrorrhizine hydrochloride (99.50%), and magnoflorine (99.82%), all purchased from Chengdu Mansite Biotechnology Co., Ltd. with batch numbers MUST-22111707, MUST-22032004, MUST-21101002, MUST23022212, MUST-21101701, MUST-22121906, MUST-22100807, MUST-21022604, MUST23022212, and MUST-22020816, respectively. Reference substances skimmianine (99.62%), tetrahydropalmatine hydrochloride (99.97%), and toddalolactone (99.50%) were purchased from Chengdu Prifa Technology Development Co., Ltd. with batch numbers PRF10081202, PRF21102042, and PRF20082821, respectively. The reference substance higenamine (97.00%, batch number Y-115-150716) was purchased from Chengdu Refines Biological Technology Co., Ltd.

1.2 Experimental Reagents and Instruments

Purified water (MS-grade, batch W6-4), methanol (MS-grade, batch A456-4), acetonitrile (MS-grade, batch A955-4), and formic acid (MS-grade, batch A117-50) were all purchased from Thermo Fisher Scientific. Instruments included UPLC-H

CLASS ultra-performance liquid chromatography, XEVO-G2 S qTOF quadrupole time-of-flight mass spectrometer, Savant™ SPD131DDA SpeedVac™ vacuum centrifugal concentrator, and MassLynx mass spectrometry data acquisition software (all from Waters Corporation, USA).

2 Experimental Methods

2.1 Preparation of Test Samples

1.0 g of the extractum of n-butanol, ethyl acetate, and alkaloid fractions from Rushanhu was weighed, dissolved in 20 mL of methanol, and 1 mL of the solution was diluted to 10 mL with methanol. The solution was filtered through a 0.22 μm membrane before injection for analysis. Reference substances (nitidine chloride, dihydrochelerythrine, chelerythrine hydrochloride, sanguinarine hydrochloride, zanthoxylin, woolly dictamnusin, dictamnine, palmatine hydrochloride, jatrorrhizine hydrochloride, magnoflorine, skimmianine, tetrahydropalmatine hydrochloride, toddalolactone, and higenamine) were each dissolved in methanol to prepare 100 μg/mL single-standard stock solutions. A 10 μg/mL mixed standard solution was prepared by diluting the single-standard stock solutions with methanol.

2.2 Chromatographic Conditions

A Waters Acquity UPLC HSS T3 column (100 mm × 2.1 mm, 1.7 μm) was used. The mobile phase consisted of 0.1% formic acid in water (A) and acetonitrile (B) with gradient elution: 0–25 min, 98%–55% A; 25–32 min, 55%–10% A; 32–32.1 min, 10%–98% A; 32.1–35 min, 98% A. The flow rate was 0.3 mL/min, the injection volume was 1.0 μL, and the column temperature was 40 °C.

2.3 Mass Spectrometric Conditions

Mass spectrometric analysis was performed using a Waters quadrupole time-of-flight mass spectrometer with an electrospray ionization (ESI) source in positive ion mode. The ESI voltage was 3 kV. The ion source temperature was 100 °C, and the desolvation temperature was 350 °C. The cone voltage was 40 kV, and the extraction

cone voltage was 4 kV. The cone gas flow rate was 50 L/h, and the desolvation gas flow rate was 600 L/h. The mass-to-charge ratio (m/z) scan range was 100–1200. Low collision energy was 10 eV, and high collision energy was 30 eV. The MSE Continuum scanning mode (non-data-dependent acquisition) was used for compound identification. The liquid chromatography-mass spectrometry system, data acquisition, and quantitative processing were controlled by MassLynx 4.1 software.

2.4 Qualitative Identification of Compounds

Raw data were acquired using the UPLC-Q-TOF MS/MS platform and imported into Progenesis QI software for peak alignment, peak extraction, and normalization, resulting in a compound list containing retention time, m/z, and peak intensity. Each ion feature was deconvoluted considering protonation and sodiation. Possible molecular formulas and candidate compounds were deduced from primary mass spectrometric data. Combined with secondary mass spectrometric fragments, fragmentation patterns of reference substances, relevant fragmentation pathways, literature, and mass spectrometric databases, the possible skeleton structures were inferred. The final molecular structures were determined by referring to reported studies on chemical components of Zanthoxylum nitidum (same family and genus as Rushanhu).

3.Result

The experiment was conducted in positive ion mode, and the primary mass spectrometry base peak maps of mixed reference substances and extracts were obtained (Figure S2). By integrating the retention time, quasi-molecular ions, secondary fragment ion information, fragmentation rules in the reference substance spectra, as well as data reported in relevant literatures, a total of 69 compounds from the alkaloids of Rushanhu (ARSH) were preliminarily screened, among which 55 were structurally identified and 14 remained unknown. The identification results are listed in Table S8. The chemical components extracted from ARSH showed close similarities,

predominantly comprising alkaloid compounds, including furoquinoline alkaloids, benzylisoquinoline alkaloids, protoberberine alkaloids, aporphine alkaloids, benzophenanthridine alkaloids, amide alkaloids, as well as other compounds such as flavonoids and coumarins.

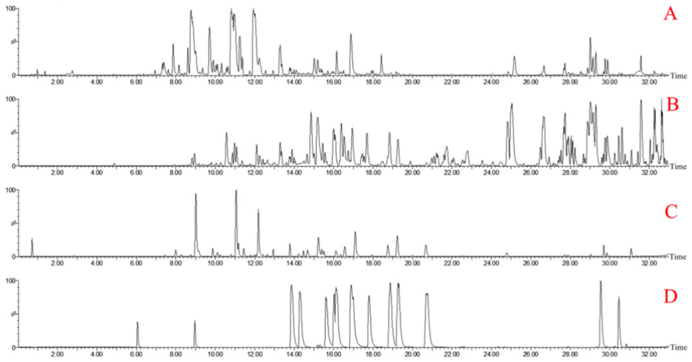

Figure. S2 Primary mass spectra of the extract and mixed reference substance of *ARSH* (A, B , C were n-butanol, ethyl acetate and alkaloid extraction parts, respectively. D was mixed standard.)

Table S8 Identification of chemical constituents in extract of Rushanhu by UPLC-Q-TOF-MS/MS

No.	T_R (min)	Selected ion	Measured mass	Calculated mass	Mass Error(mau)	Formula	Compound	Fragment ions	category	sources
1	6.06	M+H	272.1288	272.1286	0.2	$C_{16}H_{17}NO_3$	Higenamine▲	272.1288；255.1009；107.0476；161.0603	benzylisoquinoline alkaloids	Alkaloids, ethyl acetate, N-butanol extract
2	6.96	M+H	193.0941	193.0971	-3	$C_{10}H_{12}N_2O_2$	4-Methoxycinnamic acid hydrazide△	161.0665；193.0941；133.0695	phenylpropanoid compounds	Alkaloids, ethyl acetate, N-butanol extract
3	7.35	M+H	476.2488	476.2272	21.6	$C_{25}H_{33}NO_8$	3'-glucosyl-6,7-dimethoxyl-N-methyl-benzyltetrahydroisoquinoline△	314.1897；476.2488；177.0631	benzylisoquinoline alkaloids	Alkaloids, ethyl acetate, N-butanol extract
4	7.45	M+H	203.1627	203.162	0.7	$C_7H_{18}N_6O$	—*	144.0853；115.0597；203.1627	—	Alkaloids, ethyl acetate, N-butanol extract
5	7.92	M+	342.1701	342.1705	-0.4466	$C_{20}H_{24}NO_4$	Cyclanoline or Isomer	192.1086；342.1859；177.0876；148.0819	protoberberine alkaloids	Alkaloids, ethyl acetate, N-butanol extract
6	8.16	M+	342.1701	342.1705	-0.4466	$C_{20}H_{24}NO_4$	Cyclanoline or Isomer	192.1086；342.1859；177.0876；148.0819	protoberberine alkaloids	Alkaloids, ethyl acetate, N-butanol extract
7	8.67	M+	344.1857	344.1861	-0.418	$C_{20}H_{26}NO_4$	tembetarine or Isomer△	344.1988；137.0646；175.0837；299.1406	benzylisoquinoline alkaloids	Alkaloids, ethyl acetate, N-butanol extract

8	8.92	M+	342.1701	342.1705	-0.4	$C_{20}H_{24}NO_4$	Magnoflorine▲	342.1701；297.1127；265.0864	aporphine alkaloids	Alkaloids, ethyl acetate, N-butanol extract
9	9.08	M+	342.1701	342.1705	-0.4	$C_{20}H_{24}NO_4$	Magnoflorine Isomer	342.1701；297.1127；265.0864；192.1086；177.0849	aporphine alkaloids	Alkaloids, ethyl acetate, N-butanol extract
10	9.37	M+	342.1701	342.1705	-0.4466	$C_{20}H_{24}NO_4$	Cyclanoline or Isomer	177.0604；145.035；117.0392；342.1859；369.1324	protoberberine alkaloids	Alkaloids, ethyl acetate, N-butanol extract
11	9.79	M+	342.1701	342.1705	-0.4466	$C_{20}H_{24}NO_4$	Magnoflorine Isomer	342.1859；297.1232；265.0958；237.1011；205.0717	aporphine alkaloids	Alkaloids, ethyl acetate, N-butanol extract
12	9.97	M+	314.1754	314.1756	-0.2335	$C_{19}H_{24}NO_3$	Oblongine	314.1897；290.1525；269.129；218.0891	benzylisoquinoline alkaloids	Alkaloids, ethyl acetate, N-butanol extract
13	10.06	M+	358.2012	358.2018	-0.6051	$C_{21}H_{28}NO_4$	N-Methylpalaudinium△	358.2142；314.186；189.1；137.0622	benzylisoquinoline alkaloids	Alkaloids, ethyl acetate, N-butanol extract
14	10.12	M+H	249.1221	249.1233	-1.2	$C_{13}H_{16}N_2O_3$	—*	249.1221；115.0597；128.066；159.086；187.0822	amide alkaloids	Alkaloids, ethyl acetate, N-butanol extract

15	10.33	M+H	249.1221	249.1233	-1.2	$C_{13}H_{16}N_2O_3$	—*	249.1221；192.1086；187.0822；115.0597；128.066	—	Alkaloids, ethyl acetate, N-butanol extract
16	10.57	M+H	200.1282	200.1281	0.0624	$C_{10}H_{17}NO_3$	—*	200.1378；154.1285；182.1252	—	Alkaloids, ethyl acetate, N-butanol extract
17	10.7	M+	340.1544	340.1548	-0.4412	$C_{20}H_{22}NO_4$	—*	190.0946；340.1675；246.0858	aporphine alkaloids	Alkaloids, ethyl acetate, N-butanol extract
18	10.97	M+	356.1857	356.1861	-0.4363	$C_{20}H_{22}NO_5$	Menisperine or Isomer△	356.2032；279.1128；311.1395；264.0872；248.0929	aporphine alkaloids	Alkaloids, ethyl acetate, N-butanol extract
19	11.05	M+	344.1857	344.1861	-0.418	$C_{20}H_{26}NO_4$	tembetarine or Isomer△	344.1988；137.0646；175.081；299.1371	benzylisoquinoline alkaloids	Alkaloids, ethyl acetate, N-butanol extract
20	11.32	M+	328.188	328.1912	-3.1916	$C_{20}H_{26}NO_3$	4'- hydroxy- 6,7-dimethoxyl- N,N-dimethyltetrahydroisoquinoline△	328.2052；121.0691；283.1441；175.081	benzylisoquinoline alkaloids	Alkaloids, ethyl acetate, N-butanol extract
21	12.07	M+	356.1856	356.1861	-0.4886	$C_{21}H_{25}NO_4$	Menisperine or Isomer△	280.1222；356.1993；311.1395；265.0958；296.1188	aporphine alkaloids	Alkaloids, ethyl acetate, N-butanol extract

22	12.25	M+	356.1856	356.1861	-0.4886	$C_{21}H_{25}NO_4$	Menisperine or Isomer△	192.1086；356.1993；177.0849；246.0858	aporphine alkaloids	Alkaloids, ethyl acetate, N-butanol extract
23	13.29	M+H	611.2209	611.1975	23.4	$C_{28}H_{34}O_{15}$	hesperidin	611.2209；465.1591；449.1598；344.1988；303.0962	flavonoid compounds	ethyl acetate, N-butanol extract
24	13.86	M+H	356.186	356.1861	-0.1373	$C_{21}H_{25}NO_4$	tetrahydropalmatine▲	192.1044；356.1908；176.0722；165.0922	protoberberine alkaloids	Alkaloids, ethyl acetate, N-butanol extract
25	14.17	M+	322.1076	322.1079	-0.3294	$C_{19}H_{16}NO_4$	berberubine△	307.0957；322.1207；279.0991	protoberberine alkaloids	Alkaloids, ethyl acetate, N-butanol extract
26	14.27	M+	338.1396	338.1392	0.4	$C_{20}H_{20}NO_4$	jatrorrhizine▲	338.1396；322.1057；294.1118；280.0951	protoberberine alkaloids	Alkaloids, ethyl acetate, N-butanol extract
27	15.03	M+H	276.0659	276.066	-0.08	$C_{17}H_9NO_3$	liriodenine	276.0785；190.072；218.068	aporphine alkaloids	Alkaloids, ethyl acetate, N-butanol extract
28	15.22	M+H	206.0898	206.0883	1.5	$C_5H_{11}N_5O_4$	—*	206.0898；191.0663；162.0627	—	Alkaloids, ethyl acetate, N-butanol extract
29	15.31	M+H	334.1075	334.1079	-0.3899	$C_{20}H_{15}NO_4$	Dihydroavicine△	334.1185；319.0943；291.1014；206.0898	benzophenanthridine alkaloids	Alkaloids, ethyl acetate, N-butanol extract

30	15.61	M+	332.0939	332.0922	1.7	$C_{20}H_{14}NO_4$	Sanguinarine▲	332.0939；274.0867；317.0687；304.0945	benzophenanthridine alkaloids	Alkaloids, ethyl acetate, N-butanol extract
31	16.01	M+	262.1801	262.1919	-11.7883	$C_{15}H_{24}N_3O$	—*	128.066；262.1919；182.1252；189.0972	—	Alkaloids, ethyl acetate, N-butanol extract
32	16.02	M+H	309.1358	309.1338	2	$C_{16}H_{20}O_6$	Toddalolactone▲	309.1358；205.0495；235.0584；219.0644	coumarins compounds	ethyl acetate, N-butanol extract
33	16.08	M+H	264.1956	264.1963	-0.7366	$C_{16}H_{25}NO_2$	Hydroxy-α-sanshool or Isomer△	264.207	amide alkaloids	Alkaloids, ethyl acetate, N-butanol extract
34	16.12	M+	352.1551	352.1548	0.3	$C_{21}H_{22}NO$	Palmatine▲	352.1551；336.1258；308.1257；294.1118；322.1057	protoberberine alkaloids	Alkaloids, ethyl acetate, N-butanol extract
35	16.24	M+H	504.2596	504.2597	-0.1	$C_{27}H_{37}NO_8$	—*	504.2596；309.1251；459.1984；277.096	—	Alkaloids, ethyl acetate, N-butanol extract
36	16.4	M+H	264.1956	264.1963	-0.7366	$C_{16}H_{25}NO_2$	Hydroxy-α-sanshool or Isomer△	264.207；105.0733；149.1021	amide alkaloids	Alkaloids, ethyl acetate, N-butanol extract
37	16.55	M+H	264.1956	264.1963	-0.7366	$C_{16}H_{25}NO_2$	Hydroxy-α-sanshool or Isomer*	190.0946；264.207；147.0727；175.0702	amide alkaloids	Alkaloids, ethyl acetate, N-butanol extract

38	16.75		280.1908	280.2009	-10.0743			280.2009	—	Alkaloids, ethyl acetate, N-butanol extract
39	16.96	M+	348.1233	348.1235	-0.2	$C_{21}H_{18}NO_4$	Nitidine▲	348.1233；332.0901；304.0945；290.083	benzophenanthridine alkaloids	Alkaloids, ethyl acetate, N-butanol extract
40	17.7	M+	234.1579	234.16	-2.1	$C_{13}H_{20}N_3O$		234.1579；133.0695；222.1572	—	Alkaloids, ethyl acetate, N-butanol extract
41	17.79	M+	348.1233	348.1235	-0.2	$C_{21}H_{18}NO_4$	Chelerythrine▲	348.1233；332.0901；304.0945；290.083	benzophenanthridine alkaloids	Alkaloids extract
42	18.49	M+H	588.2956	588.2597	35.919	$C_{34}H_{37}NO_8$	Buesgenine△	588.3201；543.2578；309.1251；262.1919	benzophenanthridine alkaloids	Alkaloids、N-butanol extract
43	18.87	M+H	260.0928	260.0923	0.5	$C_{14}H_{13}NO_4$	Skimmianine▲	227.0588；260.0928；199.0637；184.0398；245.0668	Furoquinoline alkaloids	Alkaloids, ethyl acetate, N-butanol extract
44	19.28	M+H	230.0814	230.0812	0.261	$C_{13}H_{11}NO_3$	γ-Fagarine▲	200.0349；230.0843；215.0609；172.0391；186.0551	Furoquinoline alkaloids	Alkaloids, ethyl acetate, N-butanol extract
45	19.89	M+H	285.1002	286.098	-997.765	$C_{18}H_{11}N_3O$	7,8-Dehydrorutaecarpineor Isomer△	286.1199；271.093	quinazoline alkaloids	Alkaloids, ethyl acetate, N-butanol extract

46	20.73	M+H	200.0708	200.0706	0.2012	$C_{12}H_9NO_2$	Dictamine▲	185.0489；200.0725	Furoquinoline alkaloids	Alkaloids, ethyl acetate, N-butanol extract
47	21.21	M+H	312.1597	312.1599	-0.1745	$C_{19}H_{21}NO_3$	Ailanthamide△	147.0503；312.1726；135.0858；166.1308	amide alkaloids	Alkaloids, ethyl acetate, N-butanol extract
48	21.75	M+H	246.1853	246.1852	0.1078	$C_{16}H_{23}NO$	—*	246.1949；105.0733；145.1064；147.1223	—	ethyl acetate, N-butanol extract
49	21.99	M+H	382.1286	382.129	-0.3676	$C_{21}H_{19}NO_6$	Arnottianamide△	364.133；382.1441；292.0833；277.0585	benzophenanthridine alkaloids	Alkaloids, ethyl acetate, N-butanol extract
50	22.79	M+H	264.1956	264.1963	-0.7366	$C_{16}H_{25}NO_2$	Hydroxy-α-sanshool or Isomer△	264.207	amide alkaloids	Alkaloids, ethyl acetate, N-butanol extract
51	24.84	M+H	308.1904	308.1286	61.8	$C_{19}H_{17}NO_3$	N-Acetylanonaine△	134.0651；308.1904；116.054；144.0878	aporphine alkaloids	Alkaloids, ethyl acetate, N-butanol extract
52	25.1	M+	320.092	320.0922	-0.2129	$C_{19}H_{14}NO_4$	Coptisine△	305.0802；277.0824；320.104；248.08；219.0754	protoberberine alkaloids	Alkaloids, ethyl acetate, N-butanol extract
53	26.49	M+H	318.1238	318.0766	47.2	$C_{19}H_{11}NO_4$	Noravicine△	318.1374；302.1031	benzophenanthridine alkaloids	Alkaloids, ethyl acetate, N-butanol extract

54	26.67	M+H	288.1132	288.1136	-0.4	$C_{18}H_{13}N_3O$	Rutaecarpine△	288.124；273.1014；244.0964	quinazoline alkaloids	Alkaloids, ethyl acetate, N-butanol extract
55	27.72	M+H	383.1288	383.1283	0.5	$C_{25}H_{18}O_4$	—*	383.1288；135.0502；365.1176；165.0617；334.096	—	Alkaloids, ethyl acetate, N-butanol extract
56	27.91	M+H	286.0978	286.098	-0.2	$C_{18}H_{11}N_3O$	7,8-Dehydrorutaecarpineor Isomer△	286.1095；257.1043；167.0658	quinazoline alkaloids	Alkaloids, ethyl acetate, N-butanol extract
57	28.87	M+H	248.2011	248.2008	0.3	$C_{16}H_{25}NO$	Sanshool or Isomer△	248.2121；133.1073；149.1396；147.1223；154.1285	amide alkaloids	ethyl acetateextract
58	29.02	M+H	248.2011	248.2011	0	$C_{16}H_{25}NO$	Sanshool or Isomer△	248.2121；133.1073；149.1396；147.1223；154.1285	amide alkaloids	Alkaloids, ethyl acetate, N-butanol extract
59	29.15	M+H	248.2011	248.2011	0	$C_{16}H_{25}NO$	Sanshool or Isomer△	248.2121；133.1073；149.1396；147.1223；154.1285	amide alkaloids	Alkaloids, ethyl acetate, N-butanol extract
60	29.29	M+	288.1726	288.1712	1.4	$C_{16}H_{22}N_3O_2$	2-(4-methoxyphenyl)-N-[(1,3,5-trimethyl-4H-	232.1084；201.0884；159.0756；174.0995；288.1726	—	Alkaloids, ethyl acetate, N-butanol extract

							pyrazol-4-yl)methyl]acetamide△			
61	29.52	M+H	275.1288	275.1283	0.4904	$C_{16}H_{18}O_4$	Toddaculine▲	275.1311；217.049；205.0495；161.0603	coumarins compounds	Alkaloids, ethyl acetate, N-butanol extract
62	29.78	M+H	334.1078	334.1079	-0.1	$C_{20}H_{15}NO_4$	Norchelerythrine	334.1222；318.0899；290.0933；276.0785	benzophenanthridine alkaloids	Alkaloids, ethyl acetate, N-butanol extract
63	29.87	M+H	262.2168	262.1807	36.1164	$C_{16}H_{23}NO_2$	Hydroxy-α-sanshool or Isomer△	262.2284；204.1468；188.1136	amide alkaloids	Alkaloids, ethyl acetate, N-butanol extract
64	30.47	M+H	350.139	350.1392	-0.1943	$C_{21}H_{19}NO_4$	Dihydrochelerythrine▲	334.1068；350.142；290.083；318.0787	benzophenanthridine alkaloids	Alkaloids, ethyl acetate, N-butanol extract
65	30.63	M+H	252.2453	252.244	1.3	$C_{15}H_{29}N_3$	(5S)-5-butyl-1-[(4-methylcyclohexyl)methyl]-4,5-dihydroimidazol-2-amine△	252.2453；227.1379	amide alkaloids	Alkaloids, ethyl acetate, N-butanol extract
66	31.57	M+H	254.261	254.1175	143.5	$C_{16}H_{15}NO_2$	alatamide△	254.261；198.1943	amide alkaloids	Alkaloids, ethyl acetate, N-butanol extract

67	32.05	M+H	392.1868	392.1497	37.0979	$C_{23}H_{21}NO_5$	dihydrocherythrinylacetaldehyde△	334.1259；392.2099；348.143；360.1805	benzophenanthridine alkaloids	Alkaloids, ethyl acetate, N-butanol extract
68	32.26	M+H	495.4257			$C_{30}H_{52}N_6$	—*	495.4257；348.143；248.2154	—	ethyl acetate, N-butanol extract
69	32.63	M+H	495.4257			$C_{30}H_{52}N_6$	—*	495.4257；348.143；248.2154	—	Alkaloids、 ethyl acetate、N-butanol extract

▲represents that after comparison of reference substances, △ has literature and database reference, unmarked means no literature reference, and * is regarded as an unknown component
